# Salmonella enteritidis meningitis in a first time diagnosed AIDS patient: Case report

**DOI:** 10.1186/1757-1626-1-5

**Published:** 2008-05-12

**Authors:** Chrisostomos Katsenos, Nectarios Anastasopoulos, Maria Patrani, Costas Mandragos

**Affiliations:** 1 Red Cross Hospital of Athens Korgialeneio-Benakeio, Intensive Care Unit, Athanasaki 1 str, Ampelokipoi 11526 Athens, Greece

## Abstract

We describe a patient with salmonella enteritidis meningitis and unknown HIV infection.

Setting: A 14-bed adult intensive care unit in a tertiary hospital.

The patient was brought to the emergency department with fever, nuchal rigidity and confusion. A first cerebrospinal fluid examination was non diagnostic. After a short period of improvement the patient developed septic shock. A second cerebrospinal fluid specimen was purulent. Both specimens yielded salmonella enteritidis and a blood culture as well. An Eliza reaction was performed and showed positive for HIV. The CD4(+) cells count was 16/mm^3^. The patient died with refractory shock eight days after admission in the intensive care unit.

## Introduction

Salmonellae are motile gram-negative bacilli, which infect or colonize a wide range of mammalian hosts. In humans can cause gastroenteritis, enteric (typhoid) fever, bacteremia, absess, osteomyelitis. After entering the bloodstream all tissue and organ life is susceptable. Localization of systemic infections is associated with certain predisposed conditions (pneumonia or empyema with malignancy, diabetes, sickle cell disease-urinary tract infections with urolithiasis, structural abnormalities, immunosuppression-osteomyelitis with sickle cell disease, sickle-C disease and sickle thalassemia etc). Salmonella meningitis is a rare complication and is prevalent in infants and young children [1]. Cerebrospinal fluid studies are usually normal or reveal a mild pleocytosis, even in patients with neuropsychiatric symptoms [2]. There is also evidence that immunocompromized patients fare poorly with typhoidal infections, while HIV-infected patients are prone to more severe and complicated non typhoidal salmonellosis [2]. Such patients who are not aware of their HIV status, or are not under medical supervision, may present with their first opportunistic infection in the central nervous system. These patients appear susceptible to the following: toxoplasma encephalitis, cytomegalovirus encephalitis, neurocysticercosis, tuberculosis, cryptococcal meningitis, brain absesses secondary to staphylococcus, streptococcus, salmonella, aspergillus, nocardia, rhodococcus, or listeria. While most of these entities are less likely to occur, toxoplasma encephalitis is a condition that predominates [3]. Here we present a case of meningitis caused by salmonella enteritidis in a HIV infected patient who was unaware of his severe immune suppression. He was admitted with neuropsychiatric symptoms, fever and a non diagnostic CSF examination.

## Case Presentation

A 43-year-old man was admitted to the hospital because of fever, headache, malaise and confusion. His colleagues who brought him to the hospital reported bizarre behaviour and difficulty in communication. His temperature was 38, 5°C, blood pressure 135/70 mmHg, pulse rate 90/min, sinus rhythm. Under examination the patient was alert, but slowly reactive and disorientated. Except for stiffness of neck, the rest of his physical examination was unremarkable.

The patient was single, living with his brother and mother, and worked as a truck driver with international freight. His sexual preference is unknown. He smoked regularly one pack of cigarettes per day for 25 years, drank occasionally and smoked marijuana from time to time.

Five years ago, he was admitted to another hospital because of bilateral facial nerve palsy, which resolved. One month ago he had another bout of confusion, and for several months he had a dry cough.

A C/T of the brain was normal and a chest X-ray was unremarkable. A lumbar puncture was also performed. The CSF was clear, colorless. The cell count was as follows: Cells per mm^3^: Red 10,200, White 2(lymphocytes). Protein concentration was 45 mg/dl and glucose was 95 mg/dl, while blood glucose was 236 mg/dl. The hematocrit was 36.7%, white cell count 5700 per mm^3^, (neutrofils 69%, lymphocytes 19% monocytes 8%), platelet count: 196,000. BUN was 33 mg/dl and creatinine was 2.7 mg/dl. There were no other laboratory test results of significance.

The patient was prescribed cefuroxime and on the following day his mental status improved. He could eat and cooperate properly under physical examination. Suddenly, on the third day, fever rose to 39, 5°C the patient became comatose (GCS 7/15) and his blood pressure was 60/40 mmHg, pulse rate 85/min. Dopamine and then Dobutamine was administered. The patient was intubated, mechanical ventilation was introduced and he was transferred to the ICU.

Cefuroxime was discontinued and cefepime, vancomycin, amikacin and acyclovir were administered. A second lumbar puncture was then performed. The CSF was purulent in appearance. The cell count now was as follows: white cells 38.000(neutrofils 98%, lymphocytes 2%) protein: 1,5 gr/dl and glucose: 8 mg/dl, while blood glucose was 150 mg/dl. Microscopical examination: intracellular gram (-) rods. White cell count indicated 7100 per mm^3 ^(neutrofils 81%, lymphocytes 10%, monocytes 5%).

The culture of the first CSF specimen yielded salmonella enteritidis susceptible to ciprofloxacin, second and third generation cephalosporins. Hence ciprofloxacin was added, while acyclovir and vancomycin were discontinued. An Eliza reaction for HIV was performed and showed positive. The results were confirmed by Western-Blot reaction. The following day a blood culture also yielded Salmonella enteritidis. The CD4 (+) cells were 16/mm^3^.

On the eighth day after admission the patient died with refractory shock and multiorgan failure.

## Discussion

Salmonella meningitis is quit an unusual complication in adults, while infants and young children are more susceptible to it. All reported cases in adults are associated with immune suppression. (Cancer, Aids, immunotherapy)

Although HIV infected patients demonstrate increased susceptibility to serious infections from non typhoidal salmonellae, salmonella meningitis remains a rare complication.

To our best knowledge only 10 cases of salmonella spp meningitis have been reported worldwide in such patients [4,5].

Interestingly our patient had a positive CSF culture without CSF WBC's or elevated protein, but with a relatively low glucose concentration (less than a half of the blood concentration). Although it is not clear why the patient initially improved and then worsened, treatment seemed to slow the progression of the infection without eradicating the salmonellae. Hence, patient's profound immunosupression most likely influenced the fatal outcome.

## Conclusion

A CSF culture positive for salmonella spp. should raise the suspicion of severe immune suppression resulting from HIV infection or advanced cancer [6-8]. This infection bears a high mortality, while relapses may occur [9,10].

## List of abbreviations

C/T: computerized tomography; CSF: cerebrospinal fluid; GCS: Glasgow Coma Scale; WBC: white blood count.

## Competing interests

The authors declare that they have no competing interests.

## Authors' contributions

All authors were involved in patient's care. CK and NA prepared the manuscript. CM coordinated the presentation. All authors read and approved the final manuscript.

## Consent

Written informed consent was obtained from patient's relatives for publication of this case report
